# Q-TWiST analysis of first-line nivolumab plus chemotherapy versus chemotherapy in patients with advanced gastric cancer, gastroesophageal junction cancer, or esophageal adenocarcinoma from CheckMate 649: 4-year follow-up results

**DOI:** 10.1007/s10120-025-01634-6

**Published:** 2025-07-09

**Authors:** Daniel Lin, Wenying Quan, Marne Garretson, Viktor Chirikov, Clara Chen, Prianka Singh, Catherine Davis, Ryan Sugarman

**Affiliations:** 1 Department of Medical Oncology, Thomas Jefferson University Hospital, 1025 Walnut Street, Suite 700 College Building, Philadelphia, PA 19107 USA; 2 OPEN Health, HEOR and Market Access, 1140 6 Ave., Floor 18, New York, NY 10036 USA; 3 Bristol Myers Squibb, Route 206 and Province Line Road, Princeton, NJ 08543 USA; 4 Memorial Sloan Kettering Cancer Center, New York, NY 10065 USA

**Keywords:** Esophageal adenocarcinoma, Gastric cancer, Gastroesophageal junction cancer, Nivolumab, Q-TWiST

## Abstract

**Background:**

Nivolumab plus chemotherapy demonstrated clinically significant improvement in quality-adjusted survival versus chemotherapy alone as first-line treatment for advanced non-HER2-positive gastric cancer, gastroesophageal junction cancer, and esophageal adenocarcinoma (GC/GEJC/EAC) in the CheckMate 649 post-hoc quality-adjusted time without symptoms or toxicity (Q-TWiST) analysis at 1-year minimum follow-up. We report Q-TWiST analysis results at 4-year minimum follow-up.

**Methods:**

Q-TWiST methodology was applied post-hoc to CheckMate 649 study data from all randomized patients, patients with PD-L1 combined positive score (CPS) ≥ 1, and patients with PD-L1 CPS ≥ 5. Relative Q-TWiST gains ≥ 10% were predefined as clinically important and ≥ 15% as clearly clinically important.

**Results:**

Among all randomized patients, patients with PD-L1 CPS ≥ 1, and patients with PD-L1 CPS ≥ 5, mean (95% CI) absolute Q-TWiST gains of 3.4 (1.8–5.1), 4.2 (2.4–6.1), and 5.4 (3.0–7.7) months with nivolumab plus chemotherapy versus chemotherapy were observed, respectively. These translated to clearly clinically important relative Q-TWiST gains of 20.5%, 26.1%, and 33.4% in each population; relative Q-TWiST gains benefit remained clearly clinically important in all subgroups (15.7%, 20.3%, and 26.4%) after expanding the analysis to include grade 2 adverse events. Greater Q-TWiST gains were observed with nivolumab plus chemotherapy across most subgroups in all randomized patients and patients with PD-L1 CPS ≥ 1 and across all subgroups in patients with PD-L1 CPS ≥ 5.

**Conclusion:**

Clearly clinically important benefit in quality-adjusted survival with first-line nivolumab plus chemotherapy versus chemotherapy was observed across all evaluated PD-L1 CPS expression levels in patients with advanced GC/GEJC/EAC from CheckMate 649 with 4-year minimum follow-up.

**Trial Registration:**

ClinicalTrials.gov identifier, NCT02872116.

**Supplementary Information:**

The online version contains supplementary material available at 10.1007/s10120-025-01634-6.

## Introduction

In the CheckMate 649 study (NCT02872116), nivolumab plus chemotherapy demonstrated superior overall survival (OS) and clinically meaningful progression-free survival (PFS) benefit as assessed by blinded independent central review (BICR) versus chemotherapy alone, with an acceptable safety profile, in patients with previously untreated, advanced, non-human epidermal growth factor receptor 2 (HER2)-positive gastric cancer, gastroesophageal junction cancer, and esophageal adenocarcinoma (GC/GEJC/EAC) [1]. Based on these results, nivolumab plus chemotherapy has been approved in more than 50 countries for the first-line treatment of advanced or metastatic GC/GEJC/EAC. After 3 years of minimum follow-up, nivolumab plus chemotherapy continued to show clinically meaningful OS and PFS improvement versus chemotherapy in both the overall study population and in patients with programmed death ligand 1 (PD-L1) combined positive score (CPS) ≥ 5 [2].

Given the increasingly recognized importance of evaluating the comprehensive impact of treatment on patient survival and quality of life (QoL) [3–6], a post-hoc quality-adjusted time without symptoms or toxicity (Q-TWiST) analysis, which incorporates the quantity and quality of survival time into a single metric, was initially performed on the 1-year follow-up trial data to further understand the benefit-risk profile and quality-adjusted survival gain with nivolumab plus chemotherapy [7]. With 12 months of minimum follow-up, a mean Q-TWiST gain of 1.8 months (95% confidence interval [CI], 0.9–2.7) with nivolumab plus chemotherapy versus chemotherapy was observed in all randomized patients, representing a relative Q-TWiST gain of 12.8%; in patients with PD-L1 CPS ≥ 5, the mean Q-TWiST gain was greater (2.8 months [95% CI, 1.5–4.1], with a relative Q-TWiST gain of 20.6%) [7]. Sensitivity analyses performed across timepoints also demonstrated increased Q-TWiST gains over time in all randomized patients and in patients with PD-L1 CPS ≥ 5 [7], suggesting further increases in Q-TWiST gains might be observed with longer follow-up time. Here, we report 4-year follow-up results of Q-TWiST analyses in all randomized patients, patients with PD-L1 CPS ≥ 1, and patients with PD-L1 CPS ≥ 5.

## Materials and methods

### CheckMate 649 study methods

The methods used in the global, randomized, open-label, phase 3 CheckMate 649 study have been previously reported [1, 2].

### Data source

The post-hoc Q-TWiST analysis was conducted using trial data of all randomized patients, patients with PD-L1 CPS ≥ 1, and patients with PD-L1 CPS ≥ 5 from the CheckMate 649 study (48-month minimum follow-up; 66-month maximum follow-up; July 10, 2023, database lock).

### Statistical analyses

The Q-TWiST methodology employed has been described in detail previously [7]. Briefly, the area under the OS Kaplan–Meier curve for each treatment arm was partitioned into three distinct health states: time with toxicity before disease progression (TOX), time without symptoms or toxicity (TWiST), and time after disease progression (PROG; this alternative terminology was used in place of time after relapse [REL] for this disease state). TOX was defined as the number of days with grade 3 or 4 treatment-related adverse events before progressive disease or end of study follow-up, and Kaplan–Meier curves were constructed for TOX, PFS per BICR, and OS to estimate the mean durations of each using area under the curve. The mean duration of TWiST was calculated as the difference in the area under the PFS and TOX Kaplan–Meier curves. The mean duration of PROG was calculated as the difference in area under the OS and PFS Kaplan–Meier curves. The Q-TWiST for each treatment arm was then calculated as the sum of mean duration of each health state weighted by each state’s respective level of utility (equation: $Q-TWiST={U}_{TOX}\times TOX+{U}_{TWiST}\times TWiST+{U}_{PROG}\times PROG$), using the traditional utility values for the base case Q-TWiST analysis (*U*_*TOX*_ = 0.5, *U*_*TWiST*_ = 1.0, and *U*_*PROG*_ = 0.5). Absolute Q-TWiST gain was calculated as the difference in Q-TWiST between the nivolumab plus chemotherapy and chemotherapy arms. Relative Q-TWiST gain was calculated as the absolute Q-TWiST gain divided by the mean OS of patients in the chemotherapy arm. Relative Q-TWiST gains of 10% or greater were predefined as clinically important and gains of 15% or greater were considered clearly clinically important, based on previously established criteria [8]. Q-TWiST analyses stratified by patient subgroups of interest from CheckMate 649 as previously defined were also performed [7].

A sensitivity analysis was conducted to examine relative Q-TWiST gains at different predefined timepoints in 3-month intervals until maximum follow-up to assess changes in Q-TWiST difference between treatment arms over time. Furthermore, a sensitivity analysis with grade 2 adverse events lasting ≥ 28 days was performed, in addition to grade 3 and 4 adverse events in the definition of TOX, to determine the robustness of our base-case results. An exploratory Q-TWiST analysis was conducted using utility values for each health state calculated separately for each treatment arm using EQ-5D-3L data collected from CheckMate 649 and applying the UK scoring algorithm. Finally, the Q-TWiST results from this analysis of CheckMate 649 data were compared with Q-TWiST results reported in a systematic benchmark review by Solem et al. [9]. Briefly, the benchmark analysis included Q-TWiST data from 51 articles reporting 81 Q-TWiST comparisons for all cancers at any stage published prior to June 19, 2017; the Q-TWiST values from these studies were recalculated using standardized utility values, and relative Q-TWiST gains were calculated by adjusting for the underlying survival time to allow for comparison across studies [9]. All analyses were conducted using SAS software (SAS Institute, version 9.4) and 95% CIs were calculated via non-parametric bootstrap method (using 1000 resamples of study patients generated with replacement) to compare quality-adjusted survival times between nivolumab plus chemotherapy and chemotherapy.

## Results

### Summary of 4-year minimum follow-up clinical results

In total, 1581 patients with previously untreated advanced GC/GEJC/EAC were randomized to receive nivolumab plus chemotherapy or chemotherapy in the CheckMate 649 study; of these patients, 1297 had PD-L1 CPS ≥ 1 and 955 had PD-L1 CPS ≥ 5. With 48-month minimum follow-up, nivolumab plus chemotherapy continued to demonstrate OS benefit versus chemotherapy in all randomized patients (13.7 vs. 11.6 months; HR, 0.79 [95% CI, 0.71–0.88]), patients with PD-L1 CPS ≥ 1 (13.8 vs. 11.4 months; HR, 0.75 [95% CI, 0.67–0.85]), and patients with PD-L1 CPS ≥ 5 (14.4 vs. 11.1 months; HR, 0.70 [95% CI, 0.61–0.81]) (Supplementary Fig. 1). PFS benefit with nivolumab plus chemotherapy versus chemotherapy was also maintained in all randomized patients (7.7 vs. 6.9 months; HR, 0.80 [95% CI, 0.71–0.89]), patients with PD-L1 CPS ≥ 1 (7.5 vs. 6.9 months; HR, 0.77 [95% CI, 0.68–0.88]), and patients with PD-L1 CPS ≥ 5 (8.3 vs. 6.1 months; HR, 0.71 [95% CI, 0.61–0.82]) (Supplementary Fig. 2). Grade 3 or 4 treatment-related adverse events occurred in 60% and 45% of patients in the nivolumab plus chemotherapy and chemotherapy arms, respectively.

### Q-TWiST base-case and subgroup analyses

With 4 years of minimum follow-up, patients treated with nivolumab plus chemotherapy had longer mean durations of OS, PFS per BICR, TOX, TWiST, and PROG compared with patients who received chemotherapy (Table 1), and the mean differences were statistically significant for all health states except PROG across all evaluated PD-L1 CPS expression levels. In all randomized patients, the mean duration of Q-TWiST was 16.9 months with nivolumab plus chemotherapy versus 13.6 months with chemotherapy, corresponding to an absolute Q-TWiST gain of 3.4 months (Table 1) and relative gain of 20.5%. The TOX, TWiST, and PROG partition survival curves for all randomized patients are shown in Supplementary Fig. 3.

**Table 1:** Mean duration for each health state and Q-TWiST

	Mean duration (95% CI), months		
	Nivolumab plus chemotherapy	Chemotherapy	Difference (95% CI), months
All randomized patients			
TOX	1.6 (1.3–2.0)	0.8 (0.6–1.0)	0.8 (0.5–1.2)
TWiST	13.2 (11.8–14.5)	10.6 (9.3–11.7)	2.6 (0.9–4.4)
PROG	5.9 (4.9–6.9)	5.1 (4.3–6.1)	0.8 (− 0.6 to 2.0)
Q-TWiST	16.9 (15.7–18.2)	13.6 (12.4–14.6)	3.4 (1.8–5.1)
OS	20.7 (19.3–22.2)	16.5 (15.4–17.6)	4.2 (2.5–6.0)
PFS^a^	14.8 (13.4–16.2)	11.4 (10.1–12.5)	3.4 (1.8–5.3)
Patients with PD-L1 CPS ≥ 1			
TOX	1.7 (1.3–2.0)	0.8 (0.6–1.0)	0.9 (0.4–1.3)
TWiST	13.5 (12.1–15.0)	10.3 (9.1–11.5)	3.2 (1.3–5.3)
PROG	6.1 (5.1–7.2)	5.0 (4.1–5.9)	1.1 (− 0.4 to 2.6)
Q-TWiST	17.4 (16.0–18.8)	13.2 (12.1–14.3)	4.2 (2.4–6.1)
OS	21.3 (19.7–22.8)	16.1 (14.9–17.3)	2.5 (3.3–7.3)
PFS^a^	15.2 (13.6–16.7)	11.2 (9.8–12.4)	4.1 (2.1–6.2)
Patients with PD-L1 CPS ≥ 5			
TOX	1.7 (1.2–2.1)	0.8 (0.5–1.1)	0.8 (0.4–1.4)
TWiST	14.7 (12.9–16.6)	10.5 (9.0–12.0)	4.2 (1.7–6.6)
PROG	6.4 (5.1–7.7)	4.8 (3.9–5.9)	1.6 (− 0.2 to 3.3)
Q-TWiST	18.7 (16.9–20.4)	13.3 (11.9–14.8)	5.4 (3.0–7.7)
OS	22.7 (20.7–24.5)	16.1 (14.6–17.7)	6.6 (4.0–8.9)
PFS^a^	16.3 (14.4–18.2)	11.3 (9.7–12.9)	5.0 (2.4–7.6)

^a^Assessed by blinded independent central review

*CI* confidence interval, *CPS* combined positive score, *OS* overall survival, *PD-L1* programmed death ligand 1, *PFS* progression-free survival, *PROG* time after progression, *Q-TWiST* quality-adjusted time without symptoms or toxicity, *TOX* time with grade 3 or 4 adverse event before disease progression, *TWiST* time without disease progression or symptoms of toxicity

Patients with PD-L1 CPS ≥ 1 had a mean Q-TWiST duration of 17.4 months with nivolumab plus chemotherapy versus 13.2 months with chemotherapy, leading to a higher absolute Q-TWiST gain of 4.2 months (Table 1) and higher relative gain of 26.1% compared with all randomized patients.

Furthermore, in patients with PD-L1 CPS ≥ 5, the mean Q-TWiST duration with nivolumab plus chemotherapy was 18.7 months versus 13.3 months with chemotherapy (Table 1), resulting in the highest absolute and relative Q-TWiST gains (5.4 months and 33.4%, respectively).

**Fig. 1:**
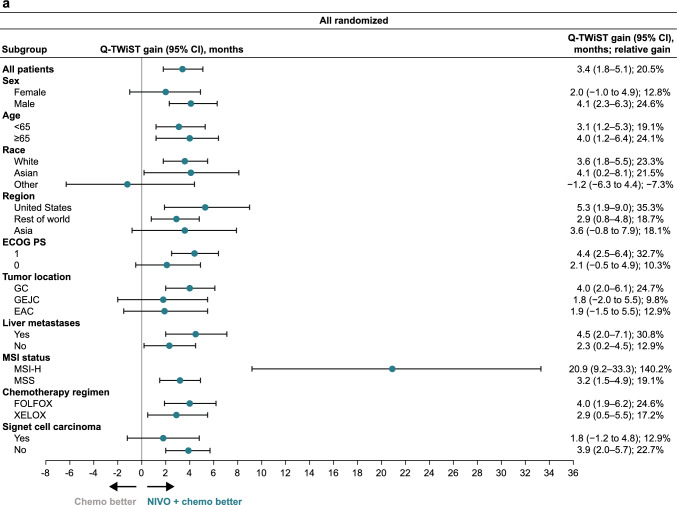

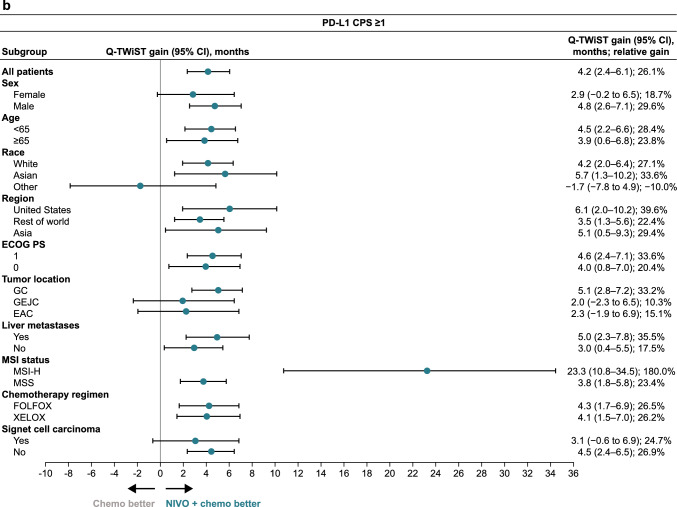

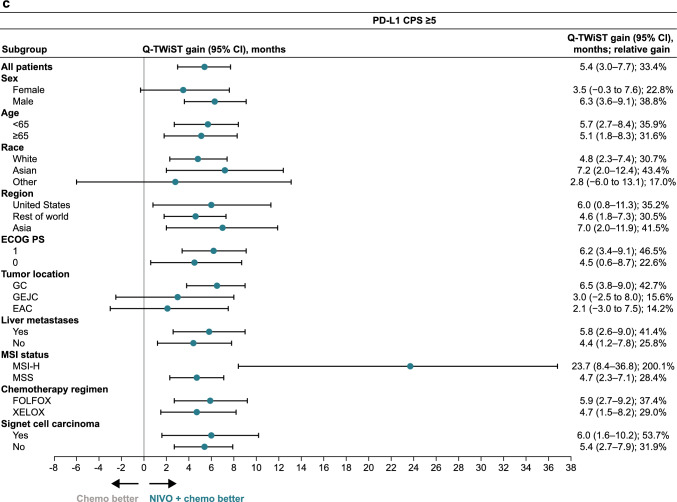
Base-case Q-TWiST subgroup analysis. **a** All randomized patients. **b** Patients with PD-L1 CPS ≥ 1. **c.** Patients with PD-L1 CPS ≥ 5. *Chemo* chemotherapy, *CI* confidence interval, *CPS* combined positive score, *EAC* esophageal adenocarcinoma, *ECOG PS* Eastern Cooperative Oncology Group performance status, *FOLFOX* leucovorin fluorouracil and oxaliplatin, *GC* gastric cancer, *GEJC* gastroesophageal junction cancer, *MSI* microsatellite instability, *MSI-H* microsatellite instability-high, *MSS* microsatellite stable, *NIVO* nivolumab, *PD-L1* programmed death ligand 1, *Q-TWiST* quality-adjusted time without symptoms or toxicity, *XELOX* capecitabine and oxaliplatin

The Q-TWiST benefit with nivolumab plus chemotherapy versus chemotherapy in all randomized patients and in patients with PD-L1 CPS ≥ 1 was observed in most prespecified subgroups (Fig. 1). Nivolumab plus chemotherapy demonstrated Q-TWiST benefit versus chemotherapy alone across all subgroups of patients with PD-L1 CPS ≥ 5 (Fig. 1). Across all randomized patients, patients with PD-L1 CPS ≥ 1, and patients with PD-L1 CPS ≥ 5, the greatest relative Q-TWiST gains were observed in the microsatellite instability-high subgroup (140.2%, 180.0%, and 200.1%, respectively).

### Q-TWiST sensitivity analyses

**Fig. 2:**
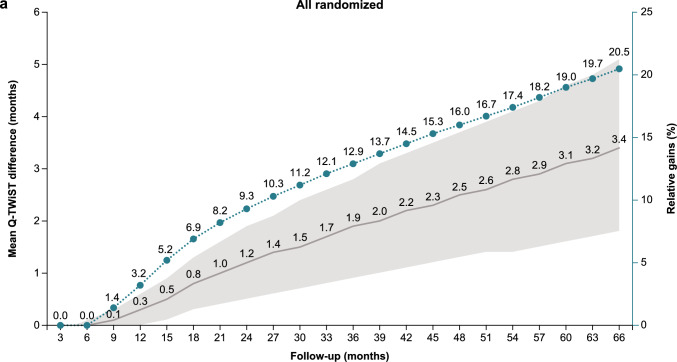

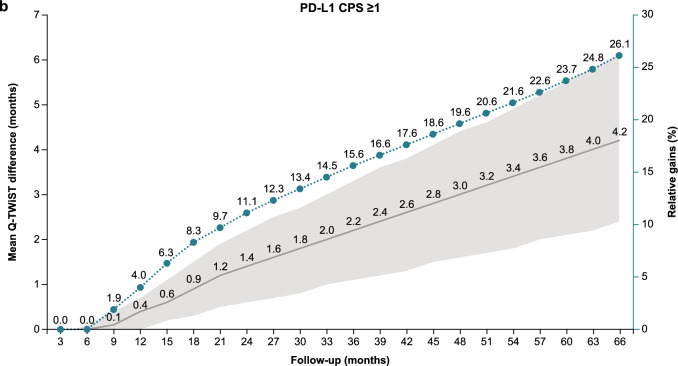

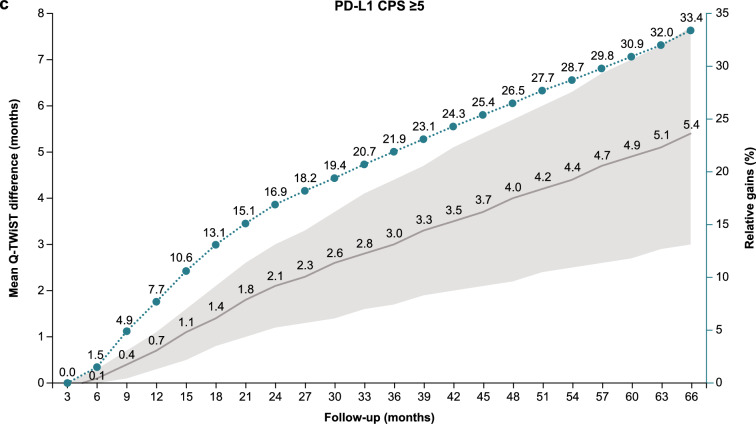
Q-TWiST sensitivity analysis. **a** All randomized patients. **b** Patients with PD-L1 CPS ≥ 1. **c** Patients with PD-L1 CPS ≥ 5. *CPS* combined positive score, *PD-L1* programmed death ligand 1, *Q-TWiST* quality-adjusted time without symptoms or toxicity

Sensitivity analysis at follow-up times from 3 to 66 months showed an increase in the relative Q-TWiST gain with longer follow-up time for all randomized patients, patients with PD-L1 CPS ≥ 1, and patients with PD-L1 CPS ≥ 5 (Fig. 2). All randomized patients had a clinically important relative Q-TWiST gain after 26 months and clearly clinically important gain after 44 months. Clinically important and clearly clinically important relative Q-TWiST gains were seen earlier in patients with PD-L1 CPS ≥ 1 (22 and 34 months, respectively). Patients with PD-L1 CPS ≥ 5 had the earliest relative Q-TWiST gains with a clinically important gain achieved after 14 months and a clearly clinically important gain after 21 months. Among all randomized patients, the duration of TWiST showed continued increase with nivolumab plus chemotherapy versus chemotherapy with longer follow-up while the duration of TOX remained stable (Supplementary Fig. 3).

In the second sensitivity analysis, the absolute Q-TWiST gains with nivolumab plus chemotherapy decreased slightly with the inclusion of grade 2 adverse events lasting ≥ 28 days (2.6, 3.3, and 4.3 months, in all randomized patients, patients with PD-L1 CPS ≥ 1, and patients with PD-L1 CPS ≥ 5, respectively). Nonetheless, relative Q-TWiST gains remained clearly clinically important (15.7%, 20.3%, and 26.4%, respectively).

### Exploratory Q-TWiST analysis with EQ-5D-3L utility values

In the exploratory analysis, Q-TWiST gain was estimated using the pre-calculated UK EQ-5D-3L utility values from CheckMate 649. The UK EQ-5D-3L utility values for nivolumab plus chemotherapy were higher compared with chemotherapy across the three cohorts, indicating a better QoL during each health state. The absolute Q-TWiST gains for nivolumab plus chemotherapy in all randomized patients, patients with PD-L1 CPS ≥ 1, and patients with PD-L1 CPS ≥ 5 were 3.8, 4.6, and 6.1 months, respectively, with corresponding relative Q-TWiST gains of 23.0%, 28.4%, and 37.6% (Table 2).

**Table 2:** Exploratory Q-TWiST analysis with EQ-5D-3L utility values

	Mean duration (95% CI), months		Difference (95% CI), months	Relative Q-TWiST gain, %
	Nivolumab plus chemotherapy^a^	Chemotherapy^b^		
All randomized patients	16.4 (15.3–17.5)	12.6 (11.7–13.4)	3.8 (2.5–5.3)	23.0
Patients with PD-L1 CPS ≥ 1	17.0 (15.6–18.1)	12.4 (11.4–13.3)	4.6 (3.0–6.2)	28.4
Patients with PD-L1 CPS ≥ 5	18.4 (16.7–19.8)	12.3 (11.1–13.5)	6.1 (4.0–7.9)	37.6

^a^For all randomized patients

*U*_TWiST_ = 0.816, *U*_TOX_ = 0.762, and *U*_PROG_ = 0.748; for patients with PD-L1 CPS ≥ 1, *U*_TWiST_ = 0.818, *U*_TOX_ = 0.761, and *U*_PROG_ = 0.752; and for patients with PD-L1 CPS ≥ 5, *U*_TWiST_ = 0.828, *U*_TOX_ = 0.804, and *U*_PROG_ = 0.769

^b^For all randomized patients, *U*_TWiST_ = 0.793, *U*_TOX_ = 0.764, and *U*_PROG_ = 0.701; for patients with PD-L1 CPS ≥ 1, *U*_TWiST_ = 0.791, *U*_TOX_ = 0.782, and *U*_PROG_ = 0.713; and for patients with PD-L1 CPS ≥ 5, *U*_TWiST_ = 0.789, *U*_TOX_ = 0.793, and *U*_PROG_ = 0.706

*CI* confidence interval, *CPS* combined positive score, *PD-L1* programmed death ligand 1,

*Q-TWiST* quality-adjusted time without symptoms or toxicity

## Discussion

To our knowledge, this updated Q-TWiST analysis of CheckMate 649 with 4 years of follow-up represents the longest follow-up time incorporated into a Q-TWiST analysis to date. Nivolumab plus chemotherapy notably demonstrated increasing Q-TWiST benefit versus chemotherapy with longer follow-up time in patients with previously untreated advanced GC/GEJC/EAC. Q-TWiST gains were observed in all randomized patients and were even greater in patients with PD-L1 CPS ≥ 1 and patients with PD-L1 CPS ≥ 5. Moreover, nivolumab plus chemotherapy provided Q-TWiST benefit across most prespecified subgroups. The relative Q-TWiST gain across all three cohorts was considered clearly clinically important based on the predefined and established threshold of ≥ 15%. In addition, the Q-TWiST gain remained clearly clinically important when the analysis was expanded to include grade 2 adverse events with a duration of ≥ 28 days, reinforcing the robustness of these findings.

Interestingly, the increase in Q-TWiST benefit with nivolumab plus chemotherapy versus chemotherapy over time was primarily driven by a relative increase in the duration of TWiST compared with the 1-year follow-up results, with differences in the mean duration of TWiST with nivolumab plus chemotherapy versus chemotherapy of 1.4 and 2.6 months at the 1-year and 4-year follow-ups, respectively, among all randomized patients [7]. Considering that median OS and PFS remained consistent across 2-, 3-, and 4-year follow-ups [7, 10, 11], the increase in TWiST duration may be explained by the additional separation observed between the PFS Kaplan–Meier curves for the nivolumab plus chemotherapy and chemotherapy groups with long-term follow-up. Meanwhile, the mean duration of TOX remained similar to the 1-year follow-up, which intuitively aligns with the incidence of adverse events not increasing with longer follow-up [2, 10]. A slight increase in the mean duration of PROG with nivolumab plus chemotherapy versus chemotherapy was also seen (0.0 and 0.8 months at the 1-year and 4-year follow-ups, respectively, in all randomized patients) [7], suggesting a continued survival benefit after disease progression.

The exploratory analysis using UK EQ-5D-3L utility values also demonstrated Q-TWiST gains with nivolumab plus chemotherapy and resulted in even greater Q-TWiST benefit compared with the base-case analysis across all three patient cohorts. The higher Q-TWiST and PROG utility coefficients with nivolumab plus chemotherapy suggest better QoL before and after disease progression in patients who received nivolumab plus chemotherapy compared with chemotherapy. Further, the TOX utility coefficients were similar for both treatment groups, indicating that the addition of nivolumab to chemotherapy did not lead to a significant decrement on patients’ QoL during time with toxicity before disease progression.

The analyses employed multiple approaches with well-established methods used across the literature [9], allowing for comparison with other Q-TWiST results. The relative Q-TWiST gain reported from this study also compared favorably with benchmark reviews for all cancers at any stage. The cumulative proportion of relative Q-TWiST gain observed in all randomized patients, patients with PD-L1 CPS ≥ 1, and patients with PD-L1 CPS ≥ 5 was greater than 88%, 92%, and 100% of the Q-TWiST gains reported in a benchmark review of all cancers at any stage, respectively [9].

There are certain limitations in this study. Grade 1 adverse events were not included in any TOX definition. Although these adverse events are milder, they may still impact patient QoL, especially if they persist for significant durations. Additionally, the Q-TWiST methodology does not differentiate between types of adverse events, so it cannot account for the extent to which various adverse events affect patients’ well-being. Finally, the assumption employed by Q-TWiST analyses that adverse events only extend until disease progression may not always hold true. Therefore, caution should be exercised when interpreting these results.

## Conclusions

In summary, with longer follow-up time, this updated Q-TWiST analysis demonstrated significant improvement in quality-adjusted survival with nivolumab plus chemotherapy versus chemotherapy in patients with previously untreated advanced, non-HER2-positive GC/GEJC/EAC, confirming the clearly clinically important benefit of this treatment. These findings provide further support for the addition of nivolumab to chemotherapy as a standard-of-care first-line treatment option for patients with non-HER2-positive advanced GC/GEJC/EAC and offer new data to assist clinical decision-making of physicians determining the ideal treatment strategy for these patients.

## Supplementary Information

Below is the link to the electronic supplementary material.

Supplementary file1 (DOCX 883 kb)

## Data Availability

Bristol Myers Squibb’s policy on data sharing may be found at https://www.bms.com/researchers-and-partners/independent-research/data-sharing-request-process.html.

## References

[1] Janjigian YY, Shitara K, Moehler M, Garrido M, Salman P, Shen L, et al. First-line nivolumab plus chemotherapy versus chemotherapy alone for advanced gastric, gastro-oesophageal junction, and oesophageal adenocarcinoma (CheckMate 649): a randomised, open-label, phase 3 trial. Lancet. 2021;398(10294):27–40. 34102137 10.1016/S0140-6736(21)00797-2 PMC8436782

[2] Janjigian YY, Ajani JA, Moehler M, Shen L, Garrido M, Gallardo C, et al. First-line nivolumab plus chemotherapy for advanced gastric, gastroesophageal junction, and esophageal adenocarcinoma: 3-year follow-up of the phase III CheckMate 649 trial. J Clin Oncol. 2024;42(17):2012–20. 38382001 10.1200/JCO.23.01601 PMC11185916

[3] European Medicines Agency. EMA Regulatory Science to 2025 - Strategic reflection. 2020. Available from: https://www.ema.europa.eu/en/documents/regulatory-procedural-guideline/ema-regulatory-science-2025-strategic-reflection_en.pdf.

[4] Schnipper LE, Davidson NE, Wollins DS, Blayney DW, Dicker AP, Ganz PA, et al. Updating the American Society of Clinical Oncology Value Framework: revisions and reflections in response to comments received. J Clin Oncol. 2016;34(24):2925–34. 27247218 10.1200/JCO.2016.68.2518

[5] Oosting SF, Barriuso J, Bottomley A, Galotti M, Gyawali B, Kiesewetter B, et al. Methodological and reporting standards for quality-of-life data eligible for European Society for Medical Oncology-Magnitude of Clinical Benefit Scale (ESMO-MCBS) credit. Ann Oncol. 2023;34(4):431–9. 36549587 10.1016/j.annonc.2022.12.004

[6] Mai TTX, Jung CY, Hyunsoon C. Utilization of quality-adjusted time without symptoms or toxicity (Q-TWiST) method in oncology: a systematic review of clinical trials. J Global Oncol. 2018;4(suppl 2):102.

[7] Lin D, Nguyen H, Shah R, Qiao Y, Hartman J, Sugarman R. Quality-adjusted time without symptoms or toxicity analysis of nivolumab plus chemotherapy versus chemotherapy alone for the management of previously untreated patients with advanced gastric cancer, gastroesophageal junction cancer, or esophageal adenocarcinoma. Gastric Cancer. 2023;26(3):415–24. 36943511 10.1007/s10120-023-01372-7 PMC10115724

[8] Revicki DA, Feeny D, Hunt TL, Cole BF. Analyzing oncology clinical trial data using the Q-TWiST method: clinical importance and sources for health state preference data. Qual Life Res. 2006;15(3):411–23. 16547779 10.1007/s11136-005-1579-7

[9] Solem CT, Kwon Y, Shah RM, Aly A, Botteman MF. Systematic review and benchmarking of quality-adjusted time without symptoms or toxicity (Q-TWiST) in oncology. Expert Rev Pharmacoecon Outcomes Res. 2018;18(3):245–53. 29402128 10.1080/14737167.2018.1434414

[10] Shitara K, Moehler M, Ajani JA, Shen L, Garrido M, Gallardo C, et al. Nivolumab plus chemotherapy vs chemotherapy as first-line treatment for advanced gastric cancer/gastroesophageal junction cancer/esophageal adenocarcinoma: 4-year follow-up of the CheckMate 649 study. Oral presentation at ASCO-GI; January 18–20, 2024; San Francisco, CA and Virtual. Abstract 306.

[11] Shitara K, Ajani JA, Moehler M, Garrido M, Gallardo C, Shen L, et al. Nivolumab plus chemotherapy or ipilimumab in gastro-oesophageal cancer. Nature. 2022;603:942–8. 35322232 10.1038/s41586-022-04508-4 PMC8967713

