# Supplementary material for: Q-TWiST analysis of first-line nivolumab plus chemotherapy versus chemotherapy in patients with advanced gastric cancer, gastroesophageal junction cancer, or esophageal adenocarcinoma from CheckMate 649: 4-year follow-up results
Source: Gastric Cancer. 2025 Jul 9;28(5):935–44. doi: 10.1007/s10120-025-01634-6 (PMC12378121; doi:10.1007/s10120-025-01634-6)

**Appendix**

This appendix has been provided by the authors to give readers additional information about their work. Supplement to:

**Q-TWiST analysis of first-line nivolumab plus chemotherapy versus chemotherapy in patients with advanced gastric cancer, gastroesophageal junction cancer, or esophageal adenocarcinoma from CheckMate 649: 4-year follow-up results**

Daniel Lin^1,^* • Wenying Quan^2^ • Marne Garretson^2^ • Viktor Chirikov^2^ • Clara Chen^3^ • Prianka Singh^3^ • Catherine Davis^3^ • Ryan Sugarman^4^

^1^ Department of Medical Oncology, Thomas Jefferson University Hospital, Philadelphia, PA 19107, USA

^2^ OPEN Health, HEOR & Market Access, 1140 6th Ave., Floor 18, New York, NY 10036, USA

^3^ Bristol Myers Squibb, Route 206 & Province Line Road, Princeton, NJ 08543, USA

^4^ Memorial Sloan Kettering Cancer Center, New York, NY 10065, USA

***Corresponding author:** Thomas Jefferson University Hospital,1025 Walnut Street, Suite 700 College Building, Philadelphia, PA 19107, USA; **e-mail address:** daniel.lin@jefferson.edu

Table of Contents

[Figures 2](#_Toc198564636)

[**Supplementary Fig. 1. Overall survival results from 4-year follow-up of CheckMate 649** 2](#_Toc198564637)

[**Supplementary Fig. 2. Progression-free survival results from 4-year follow-up of CheckMate 649** 4](#_Toc198564638)

[**Supplementary Fig. 3. Partition curves for TOX, TWiST, and PROG from 4-year follow-up** 5](#_Toc198564639)

# Figures

## **Supplementary Fig. 1. Overall survival results from 4-year follow-up of CheckMate 649**

**a** All randomized patients. **b** Patients with PD-L1 CPS ≥1. **c** Patients with PD-L1 CPS ≥5. *Chemo* chemotherapy, *CI* confidence interval, *CPS* combined positive score, *NIVO* nivolumab, *OS* overall survival, *PD-L1* programmed death ligand 1


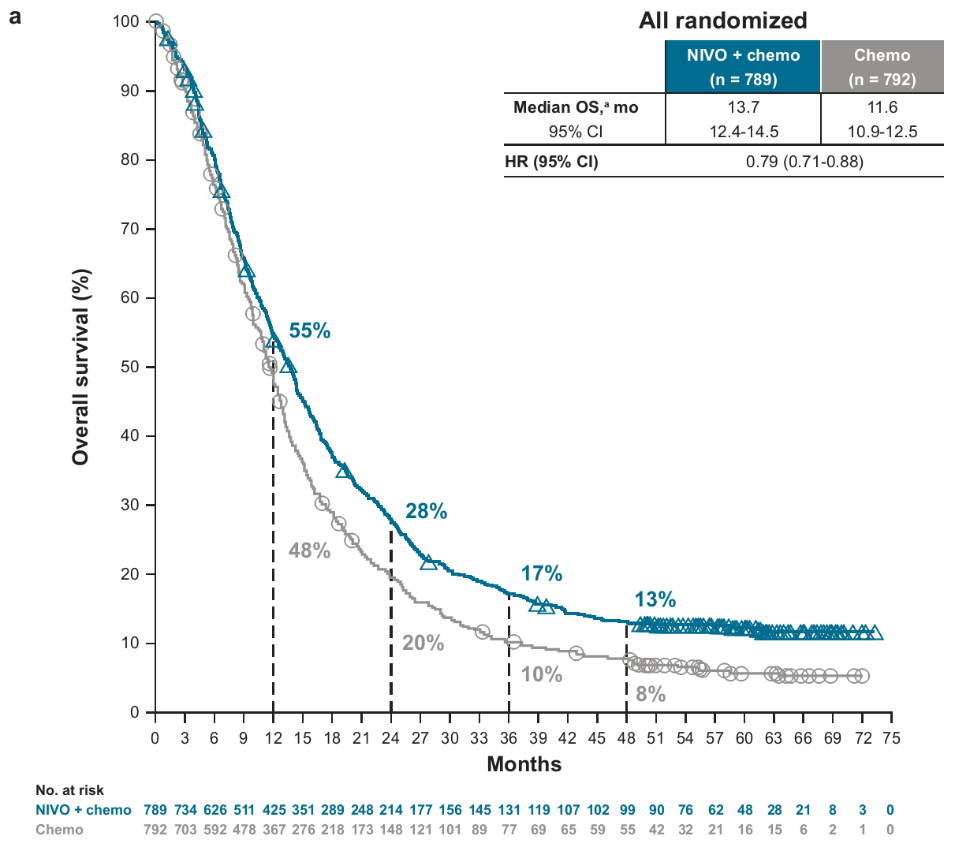


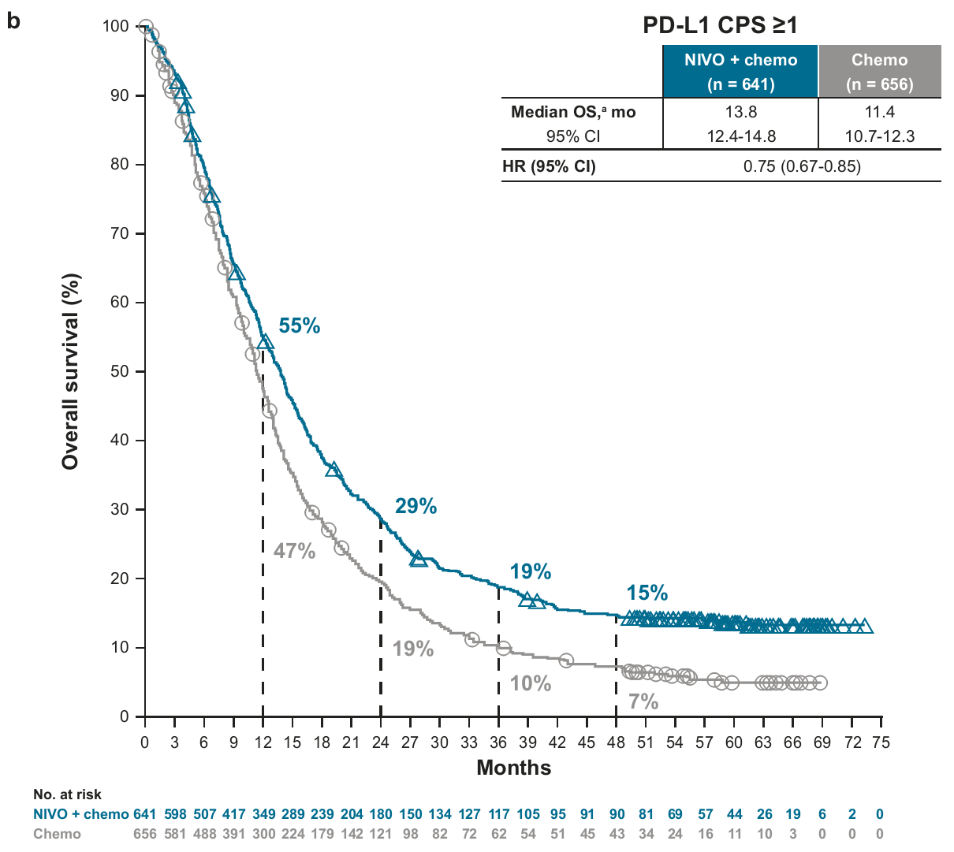


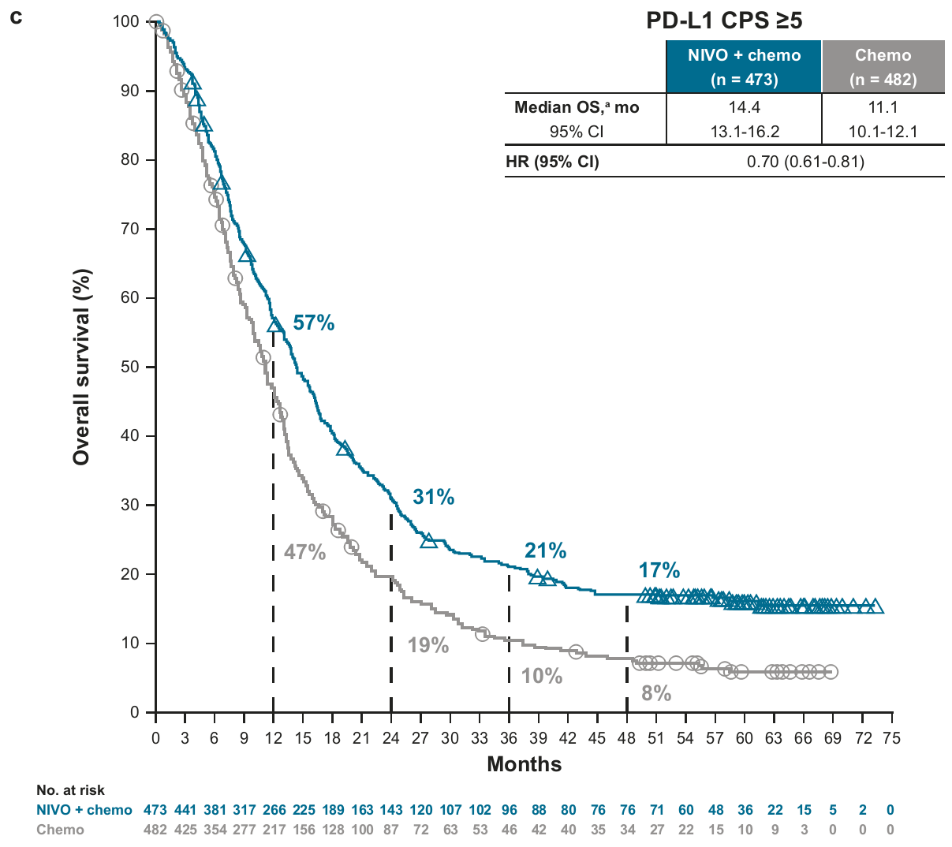


## **Supplementary Fig. 2. Progression-free survival results from 4-year follow-up of CheckMate 649**

**a** All randomized patients. **b** Patients with PD-L1 CPS ≥1. **c** Patients with PD-L1 CPS ≥5. ^a^PFS per BICR assessment. *BICR blinded independent central review,* *Chemo* chemotherapy, *CI* confidence interval, *CPS* combined positive score, *NIVO* nivolumab, *PFS* progression-free survival, *PD-L1* programmed death ligand 1


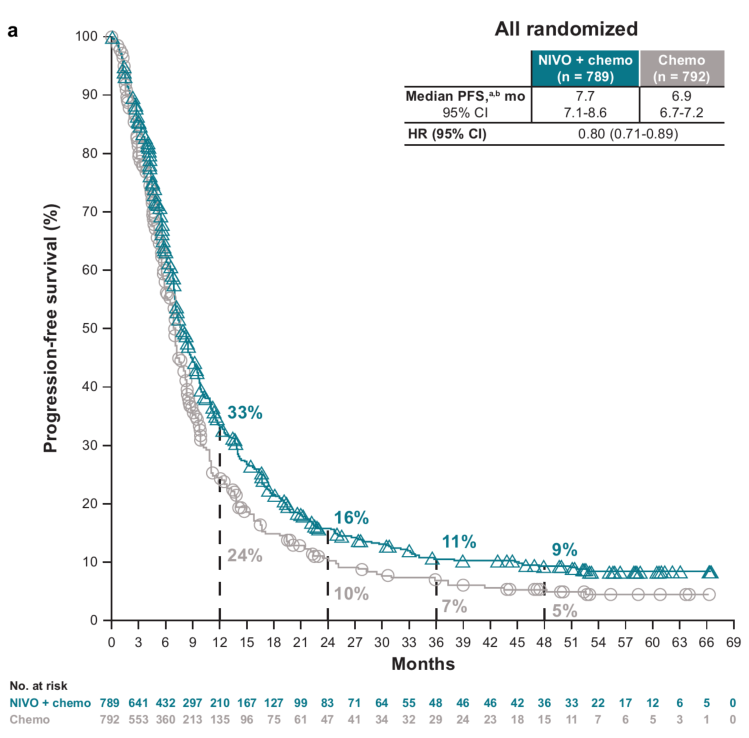


**
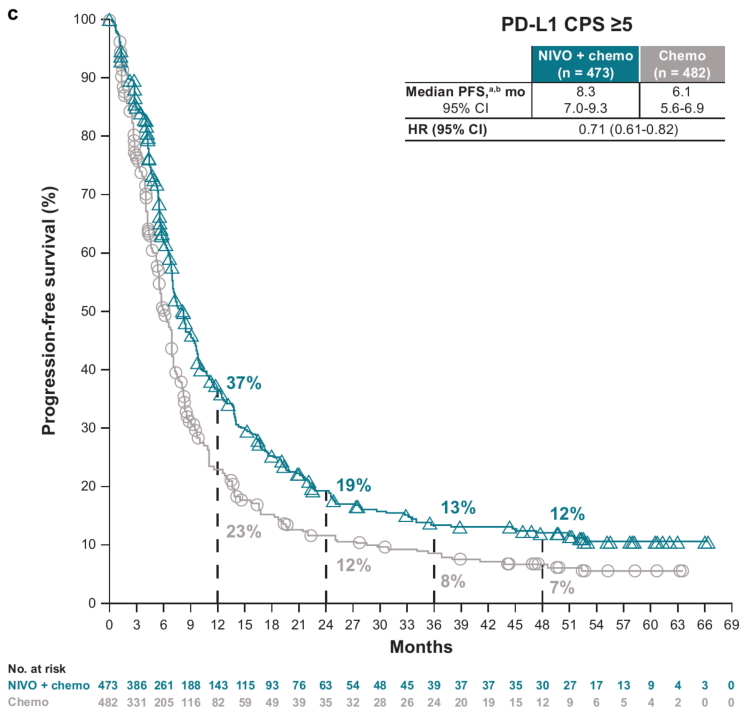
**
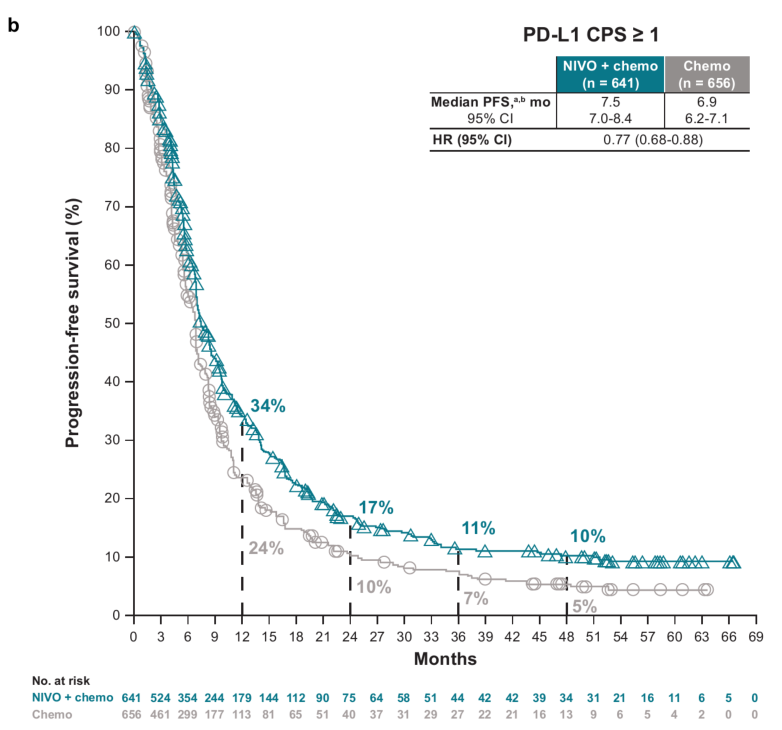


## **Supplementary Fig. 3. Partition curves for TOX, TWiST, and PROG from 4-year follow-up**

**a** NIVO + chemo. **b** chemo. *Chemo* chemotherapy, *NIVO* nivolumab.


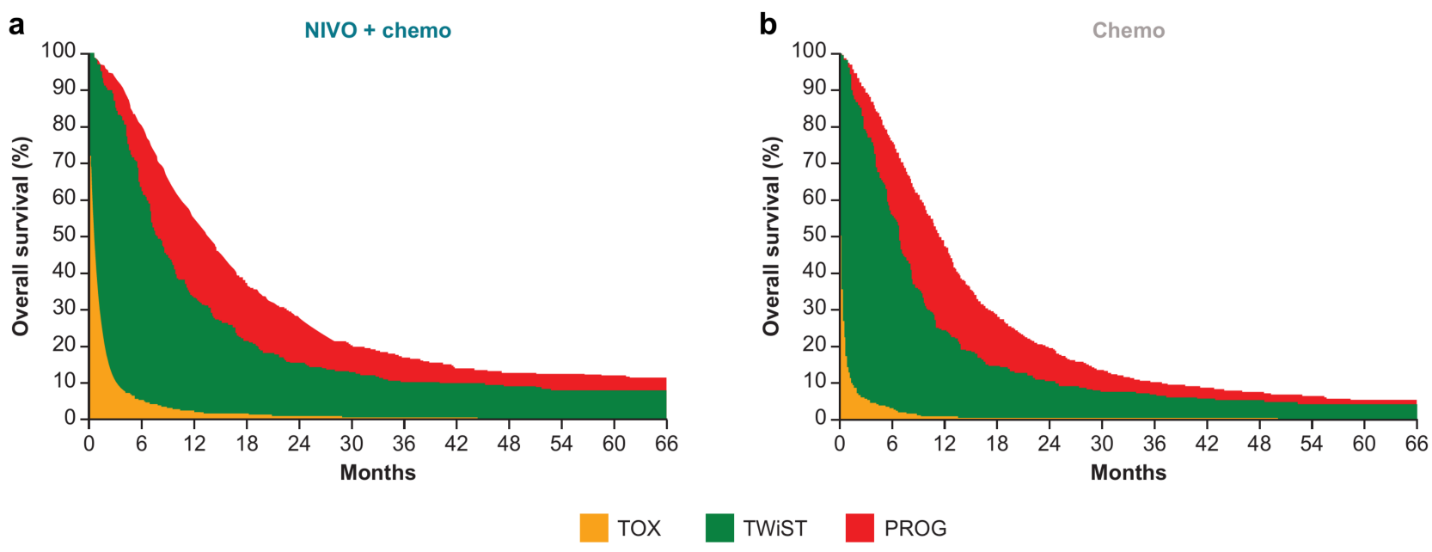

Supplement: Supplementary file 1 — Supplementary file1 (DOCX 883 kb) [file 10120_2025_1634_MOESM1_ESM.docx]
